# Dual Systems for Spatial Updating in Immediate and Retrieved Environments: Evidence from Bias Analysis

**DOI:** 10.3389/fpsyg.2018.00085

**Published:** 2018-02-06

**Authors:** Chuanjun Liu, Chengli Xiao

**Affiliations:** ^1^Department of Psychology, School of Social and Behavioral Sciences, Nanjing University, Nanjing, China; ^2^Department of Psychology, School of Social Sciences, Tsinghua University, Beijing, China; ^3^Department of Psychology, School of Law, Southwest University of Science and Technology, Mianyang, China

**Keywords:** spatial updating, spatial memory, perspective taking, alignment effect, systematic bias

## Abstract

The spatial updating and memory systems are employed during updating in both the immediate and retrieved environments. However, these dual systems seem to work differently, as the difference of pointing latency and absolute error between the two systems vary across environments. To verify this issue, the present study employed the bias analysis of signed errors based on the hypothesis that the transformed representation will bias toward the original one. Participants learned a spatial layout and then either stayed in the learning location or were transferred to a neighboring room directly or after being disoriented. After that, they performed spatial judgments from perspectives aligned with the learning direction, aligned with the direction they faced during the test, or a novel direction misaligned with the two above-mentioned directions. The patterns of signed error bias were consistent across environments. Responses for memory aligned perspectives were unbiased, whereas responses for sensorimotor aligned perspectives were biased away from the memory aligned perspective, and responses for misaligned perspectives were biased toward sensorimotor aligned perspectives. These findings indicate that the spatial updating system is consistently independent of the spatial memory system regardless of the environments, but the updating system becomes less accessible as the environment changes from immediate to a retrieved one.

## Introduction

Updating and memorizing spatial relations are extremely important in everyday life. As mobile organisms, humans evolve a powerful spatial updating system, which enables them to efficiently navigate their immediate environment, by keeping track of object locations, reaching targets, and avoiding obstacles. The spatial memory system also enables them to store and retrieve spatial information of a familiar but non-immediate environment, which is crucial in spatial activities such as planning routes to get home. The involvement of the updating-memory dual systems in spatial cognition has been generally accepted and proposed in several recent influential theories (Sholl, 2001; Wang and Spelke, 2002; Mou et al., 2004; Burgess, 2006; Waller and Hodgson, 2006; Avraamides and Kelly, 2008).

Despite the updating and memory systems being separately defined, they can work together to enable people to efficiently navigate either immediate or retrieved environments (Mou et al., 2004; Kelly et al., 2007; Avraamides et al., 2013; Greenauer et al., 2013; Hatzipanayioti et al., 2014; Xiao and Liu, 2014; Xiao et al., 2015; Liu et al., 2016; Santoro et al., 2017a,b). For example, in Kelly et al. (2007), participants learned object locations in their surrounding environment from a fixed learning direction and then either stayed in the learning room or walked to a neighboring room to perform the subsequent spatial pointing test. Participants who stayed in the learning room were blindfolded and asked to self-rotate to face a novel direction (immediate condition), those who walked to a neighboring room were asked to imagine themselves standing at the learning location and facing the learning direction, from where they retrieved their memory of the learned environment. They then self-rotated to face a novel direction (retrieved condition). After that, participants in both conditions judged target locations from three imagined perspectives, which were (1) aligned with their learning direction (memory aligned), (2) aligned with their facing direction (sensorimotor aligned), or (3) misaligned with both the learning and facing directions (misaligned). In both the immediate and retrieved conditions, participants showed memory and sensorimotor alignment effect, that is, they performed better from memory aligned and sensorimotor aligned perspectives than from misaligned perspectives. Memory alignment effect is attributed to orientation-specific representation in the memory system. As participants learned the layout from a fixed direction, their spatial memory was established with a reference direction parallel to the learning direction, from where participants can retrieve spatial representation more easily than from other novel perspectives (e.g., Shelton and McNamara, 2001). Sensorimotor alignment effect, however, is attributed to the spatial updating system, which enables people to automatically and effortless keep track of surrounding object locations with respect to themselves (e.g., Rieser, 1989; Farrell and Robertson, 1998; Farrell and Thomson, 1998; Klatzky et al., 1998). As participants rotated themselves to a novel direction, their sensorimotor cue provided by self-rotating, enabled them to perceive their current heading direction. Thus, it is relatively easy for participants to make spatial judgment from the perspective aligned with their current facing direction than from other novel directions.

Updating processes in immediate and retrieved environments are hypothesized differently. In the immediate environment, the spatial updating system is directly supported by sensorimotor/perceptual input. Idiothetic information conveys the necessary cues to monitor all relevant spatial changes (Rieser, 1989; Farrell and Thomson, 1998; Avraamides et al., 2013). If the environment is retrieved from memory (e.g., participants were either directly translated to the neighboring room or disoriented as in the studies of Kelly et al. (2007) and Xiao et al. (2015), or they were asked to remember a described environment as in the studies of Avraamides and Kelly (2010) and Avraamides et al. (2013), it cannot be updated unless it is linked to the sensorimotor framework by the physical movement (Avraamides and Kelly, 2008; Hatzipanayioti et al., 2014; Santoro et al., 2017a,b).

In parallel, results of previous studies seemed to suggest that the updating and memory systems were employed differently in immediate and retrieved environments. In the immediate environment, pointing latencies seem to be equivalent between memory aligned and sensorimotor aligned perspectives, whereas in the retrieved environment, pointing latencies in sensorimotor aligned perspective seem to be longer than that in memory aligned perspectives (e.g., Kelly et al., 2007). If the updating system keeps track of surrounding object locations with respect to the observer, these representations should be accessed very quickly or at least as fast as those retrieved from the memory system (as demonstrated in the immediate environment)^1^.

Two alternative explanations may account for the above mentioned controversy. First, the updating system maintains representation of the surrounding environment, but the representation is more deteriorated if it is updated from a retrieved representation than from a perceived representation. It is may be more difficult to access a deteriorated updated representation than a retrieved representation, resulting in longer pointing latency from the sensorimotor aligned than from the memory aligned perspective. Nevertheless, the deteriorated updated representation is still better than the computed one (i.e., the representation from the misaligned perspective), leading to a sensorimotor alignment effect. Second, the updating system only works efficiently in the immediate environment but fails to work in the retrieved environment; thus, participants have to compute the retrieved memory to locate objects from the sensorimotor aligned perspective, resulting in slower pointing from the sensorimotor aligned than from the memory aligned perspective. The sensorimotor alignment effect is attributed to the knowledge of facing direction, which enables participants to compute from the sensorimotor aligned perspective more quickly and accurately than from the misaligned perspective.

The two alternative hypotheses mentioned above could be examined by analyzing pointing bias among the three imagined perspectives. Previous studies have shown that if a spatial representation is original, there will be no bias when retrieving it. However, if a representation is transformed from the original one, it will systematically bias toward the original one (Huttenlocher et al., 1991; May, 2004; Street and Wang, 2014). Therefore, if the updating system only retains knowledge of facing direction and its representation is transformed from the retrieved memory, its representation will bias toward the memory aligned perspective. In contrast, if the updating system keeps track of the surrounding environment, this updated original representation will not bias toward the memory aligned perspective. Similarly, the transformed representation of the misaligned perspective will bias toward its original representation. If the bias is toward the sensorimotor aligned perspective, it will support the originality of the representation in the updating system.

In the present study, the process of dual systems was examined with the classical three-imagined-perspectives paradigm. The signed pointing error of each perspective was subjected to bias analysis. Participants learned the locations of eight surrounding objects, and then either remained in the learning location (the immediate environment conditions), were directly translated to or were disoriented before being escorted to the neighboring room (retrieved environment conditions)^2^. In each environment, they were asked to retrieve memory from the learning perspective, and then were asked to self-rotate 90° to face a novel perspective, from where they located targets from three imagined perspectives (i.e., memory aligned, sensorimotor aligned, and misaligned). Traditional analysis of pointing latency and absolute pointing error of each perspective was conducted to verify the memory and sensorimotor alignment effects, and to compare the relative superiority between memory and sensorimotor aligned perspectives. Importantly, signed errors of each perspective were analyzed to examine bias among perspectives.

## Materials and Methods

### Participants

One hundred and twenty university students (60 men and 60 women with normal or corrected-to-normal vision; age range = 19–26 years) participated in this experiment for monetary compensation. The ethics committee of psychology research of Nanjing University approved the study. Written informed consent was obtained from each participant before the experiments began. Four participants (two in the translated and two in the retrieved environment) were excluded from data analysis because their absolute pointing error were farther than three standard deviations from the mean.

### Materials

**FIGURE 1:**
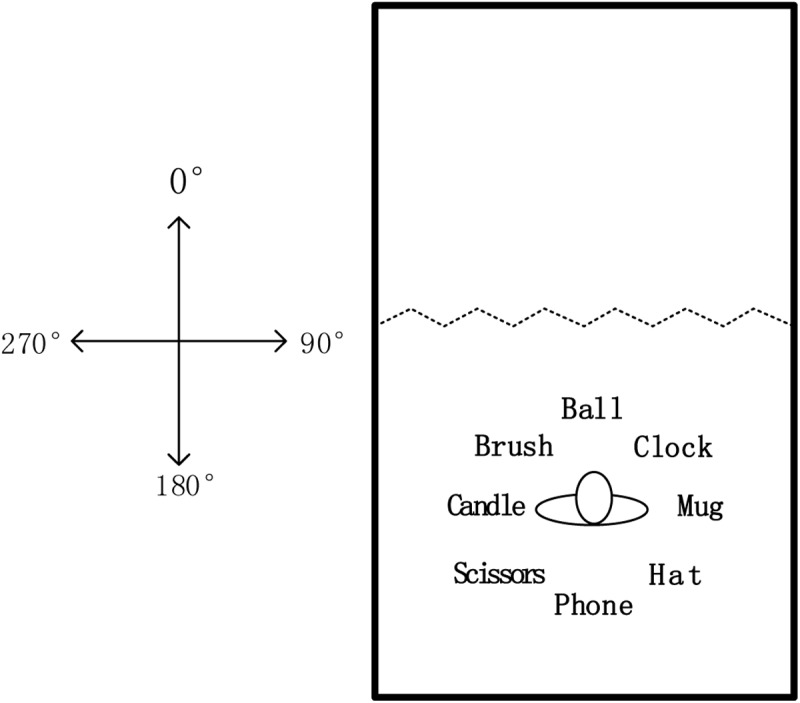
The map of object arrays in the test room for this study.

Participants learned a spatial layout consisting of eight common objects presented in a 6.5 m × 4.3 m room. As shown in **Figure 1**, 12 wooden screens separated the room into two areas. Each screen measured 0.5 m in width and 2 m in height. In the learning area, eight common objects were placed on the floor, evenly spaced. Each object was 1 m away from the center of the layout.

Test instructions, consisting of an imagined heading instruction (e.g., “Imagine you are facing the ball”) and a target-pointing instruction (e.g., “Please point to the scissors”), were presented via a wireless earphone connected to a computer. A joystick, connected to the computer, recorded the responses (pointing latency and absolute pointing error) of participants.

### Procedure and Design

Participants were assigned to one of three test environments: immediate, translated, or disoriented. In each environment, 20 males and 20 females were tested individually. They were initially familiarized with the task and joystick in several practice trials in a preparation room. The objects used in practice trials were different from those in the formal experiment. The participants practiced without limit until they had fully understood the task. Indeed, all the participants understood the task procedure after 5–10 trials. After that, they were blindfolded outside the experiment room and then were led to the middle of the layout, facing the ball. After removing the blindfold, participants could see eight target objects on the floor, and their names were given by the experimenter as the participants saw the objects with the first glance. Then with eyes closed, participants were asked to name and point to objects in any order they preferred. This learning-pointing session was repeated 10 times. Then, participants of all groups wore the blindfold again. The participants in the immediate environment stood still in the initial place. While in the translated environment group, the screen located in front of the participants was opened; then with the help of the experimenter, participants walked straight forward and stopped in the middle of the neighboring novel environment. After that, the screen was closed behind them and then participants took off the blindfold to inspect the environment. Then they were blindfold again. Whereas in the disoriented environment group, the experimenter guided the blindfolded participants around the room in a meandering walk until they were disoriented (according to the criteria of the absolute pointing error to the door at an angle greater than 90°). The meandering walk path was the same for every participant in the disoriented group similar to that used in Waller and Hodgson (2006) and Xiao and Liu (2014).

After that, participants in all groups were instructed to imagine themselves still standing at the learning position and facing the learning direction. Then, participants of all groups were asked to turn left/right to face an object (counterbalanced). This object was either a candle or mug. While facing the novel direction, participants pointed to target objects from three imagined perspectives that were either aligned with the learning direction (memory aligned), their physical facing direction during the test (sensorimotor aligned), or misaligned with the two directions mentioned above (misaligned). For example, a participant who turned to face the mug during the test would be asked to imagine himself/herself facing the ball (memory aligned perspective), the mug (sensorimotor aligned perspective), or the candle (misaligned perspective). In each imagined perspective, participants pointed to all eight objects twice. The trials were presented in a pseudo-random order with the constraint that the imagined facing objects in adjacent trials were not the same.

In accordance with previous studies, *pointing latency*, measured as the time from presentation of the name of the target object to the pointing response, and *absolute pointing error*, measured as the absolute angular difference between the guessed pointing direction and the correct direction of the target, were included in the analysis. Moreover, the *signed pointing error* for each perspective was computed with circular statistics, which indicated constant bias of pointing.

## Results

### Pointing Latency

**FIGURE 2:**
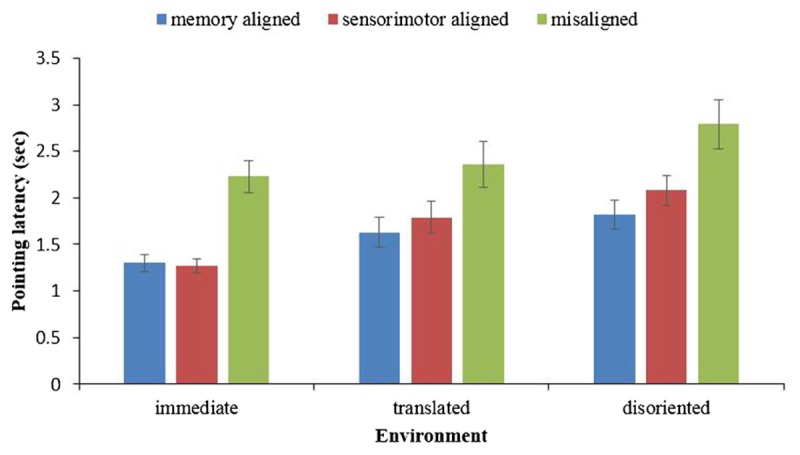
Pointing latency in immediate, translated, and disoriented environments, as a function of imagined perspective. Error bars are confidence intervals corresponding to ±1 SEM.

The pointing latency in each environment was subjected to a one-way analysis of variance (ANOVA), with the imagined perspective (memory aligned, sensorimotor aligned, misaligned) as the within-subject variable. As shown in **Figure 2**, in all three environments, the main effect of heading was significant [immediate: *F*(2,78) = 43.27, *p* < 0.001, $\eta_{p}^{2}$ = 0.53; translated: *F*(2,74) = 14.71, *p* < 0.001, $\eta_{p}^{2}$ = 0.28; and disoriented: *F*(2,74) = 17.79, *p* < 0.001, $\eta_{p}^{2}$ = 0.33]. Replicating previous findings, pairwise comparisons demonstrated memory alignment effects (the advantage of imagining from a memory aligned perspective over imagining from a misaligned perspective) and sensorimotor alignment effects (the advantage of imagining from a sensorimotor aligned perspective over imagining from a misaligned perspective) in all three environments. The memory alignment effects for immediate, translated, and disoriented environments were *F*(1,39) = 63.38, *p* < 0.001, $\eta_{p}^{2}$ = 0.62, *F*(1,37) = 25.38, *p* < 0.001, $\eta_{p}^{2}$ = 0.41, *F*(1,37) = 27.48, *p* < 0.001, $\eta_{p}^{2}$ = 0.43, respectively, and the sensorimotor alignment effect for immediate, translated, and disoriented environments were *F*(1,39) = 46.30, *p* < 0.001, $\eta_{p}^{2}$ = 0.54, *F*(1,37) = 11.32, *p* < 0.005, $\eta_{p}^{2}$ = 0.23 and *F*(1,37) = 14.06, *p* = 0.001, $\eta_{p}^{2}$ = 0.28, respectively. Moreover, the contrasts showed that the pointing latency of the memory aligned perspective was significantly shorter than that of the sensorimotor perspective in the disoriented environment [*F*(1,37) = 4.581, *p* = 0.039, $\eta_{p}^{2}$ = 0.11], but these two were not significantly different in either the immediate environment [*F*(1,39) = 0.065, *p* = 0.799, $\eta_{p}^{2}$ = 0.002], or the translated environment [*F*(1,37) = 2.549, *p* = 0.119, $\eta_{p}^{2}$ = 0.064].

### Absolute Error

**FIGURE 3:**
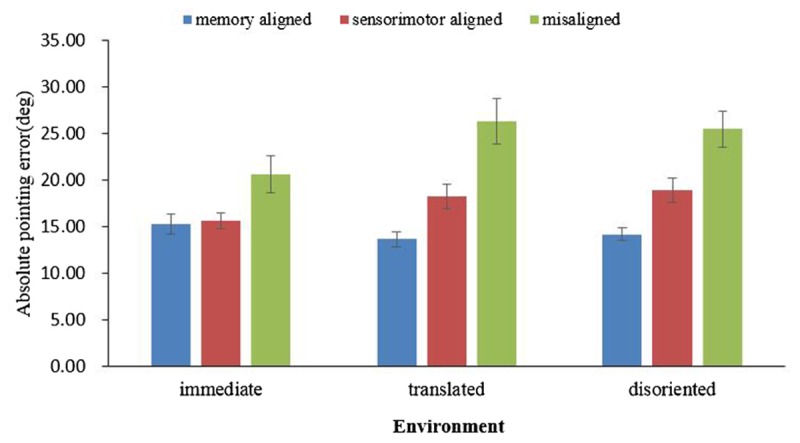
Absolute pointing error in immediate, translated, and disoriented environments, as a function of imagined perspective. Error bars are confidence intervals corresponding to ±1 SEM.

The absolute error in each environment was subjected to a one-way ANOVA, with the imagined perspective (memory aligned, sensorimotor aligned, misaligned) as the within-subject variable. As shown in **Figure 3**, in all three environments, the main effect of heading was significant [immediate: *F*(2,78) = 6.71, *p* = 0.002, $\eta_{p}^{2}$ = 0.15, translated: *F*(2,74) = 19.711, *p* < 0.001, $\eta_{p}^{2}$ = 0.35, and disoriented: *F*(2,74) = 21.861, *p* < 0.001, $\eta_{p}^{2}$ = 0.37]. Replicating previous findings, pairwise comparisons demonstrated memory and sensorimotor alignment effects in all three environments. The memory alignment effects for the immediate, translated, and disoriented environments were *F*(1,39) = 8.925, *p* = 0.005, $\eta_{p}^{2}$ = 0.19, *F*(1,37) = 34.21, *p* < 0.001, $\eta_{p}^{2}$ = 0.48 and *F*(1,37) = 38.203, *p* < 0.001, $\eta_{p}^{2}$ = 0.51, respectively, and the sensorimotor alignment effects for the immediate, translated, and disoriented environments were *F*(1,39) = 7.195, *p* = 0.011, $\eta_{p}^{2}$ = 0.16, translated, *F*(1,37) = 11.057, *p* = 0.002, $\eta_{p}^{2}$ = 0.23 and *F*(1,37) = 11.686, *p* = 0.002, $\eta_{p}^{2}$ = 0.24, respectively. Moreover, the contrasts showed that the absolute error of the memory aligned perspective was significantly smaller than that of the sensorimotor perspective in the translated environment [*F*(1,37) = 10.934, *p* = 0.002, $\eta_{p}^{2}$ = 0.228], and in the disoriented environment [*F*(1,37) = 12.347, *p* = 0.001, $\eta_{p}^{2}$ = 0.25], but these two were not significantly different in the immediate environment [*F*(1,39) = 0.083, *p* = 0.775, $\eta_{p}^{2}$ = 0.002].

Combining the results of pointing latency and absolute error, both the memory and sensorimotor alignment effects existed in the immediate and retrieved environments. The contrasts between the memory aligned perspective and the sensorimotor aligned perspective revealed that the updating system became less accessible as the environment changed from immediate to a retrieved environment.

### Circular Analysis of Signed Errors

The facing direction was counterbalanced across participants (i.e., half the participants facing 90° to the mug and the other half facing 270° to the candle), so that the sequence of memory-sensorimotor-misaligned perspectives was clockwise in the group of facing 90° but counterclockwise in the group of facing 270°. Before collapsing data of the two facing groups, the signed errors of the group of facing 270° were multiplied by -1 to reverse the direction. Then, the signed errors for each imagined perspective of each environment were subjected to one sample *t*-tests with a test value of 0 which means no signed error.

**FIGURE 4:**
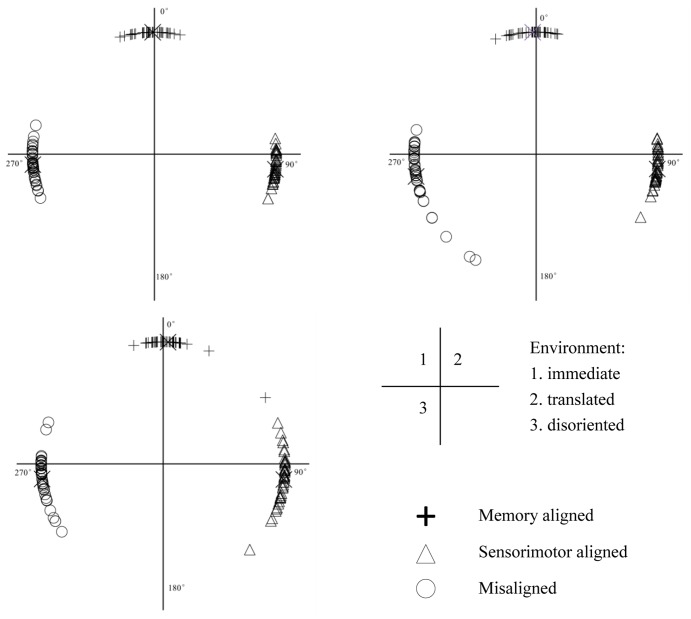
Circular distribution of signed errors of memory aligned, sensorimotor aligned and misaligned perspectives in immediate, translated, and disoriented environments. X represents the mean signed error of each perspective.

As shown in **Figure 4**, across the three environments, the signed errors of the memory aligned perspective were not biased [immediate: *t*(39) = -1.086, *p* = 0.284, 95% confidence interval (CI) = [-3.794, 1.143], translated: *t*(37) = -1.180, *p* = 0.246, CI = [-3.600, 0.951] and disoriented: *t*(37) = -0.572, *p* = 0.571, CI = [-1.589, 2.841]]. In contrast, the signed errors of the sensorimotor aligned perspective were positively biased in all three environments [immediate: *t*(39) = 6.652, *p* < 0.001, CI = [4.961, 9.297], translated: *t*(37) = 5.217, *p* < 0.001, CI = [4.260, 9.671] and disoriented: *t*(37) = 3.845, *p* < 0.001, CI = [3.248, 10.483]], and those of the misaligned perspective were negatively biased across the three environments [immediate: *t*(39) = -3.254, *p* = 0.002, CI = [-10.502, -2.451], translated: *t*(37) = -3.925, *p* < 0.001, CI = [-15.525, -4.953] and disoriented: *t*(37) = -4.577, *p* < 0.001, CI = [-10.741, -4.149]]. These results suggest that the memory and updating systems worked consistently regardless of the environment. The representation in the memory system is original while that in the updating system is not transformed from that in the memory system. The representation from the misaligned perspective is more likely transformed from that in the updating system than from that in the memory system. The two latter findings together suggest that the representation in the updating system is original in both the immediate and retrieved environments.

## Discussion

With the three-imagined-perspective paradigm, the present study replicates previous findings of memory and sensorimotor alignment effects, confirming that the two systems are employed when updating either the immediate or retrieved environments. Further, the patterns of signed errors bias indicate that the spatial updating system is independent of the spatial memory system and the updated representation is original regardless of the environment.

However, this original representation in the updating system might vary in accessibility across environment, as the absolute error and pointing latency of sensorimotor aligned perspective is equivalent to those of memory aligned perspective in the immediate environment but inferior in retrieved environments. Previous studies have shown an asymmetrical accessibility of spatial information in different egocentric directions, with privilege of objects in front of the body (Sholl, 1987; Franklin and Tversky, 1990; Werner and Schmidt, 1999). Thus, in order to further understand accessibility to updated representations at different egocentric directions, the pointing latency and absolute error of memory and sensorimotor aligned perspectives were split by pointing directions (front: objects located at an angle of 315°, 0°, and 45°; side: objects located at an angle of 90° and 270°; back: objects located at an angle of 135°, 180°, and 225°). As shown in **Table 1**, planned contrasts indicated that across environments, it is equivalent to access the front part of representations in updating and memory systems, while the back part of the representation in the updating system is consistently more difficult to access than those in the memory system. However, the relative accessibility to the side objects between the two representations vary across the environments. In the immediate environment, the updating system is more quickly accessed than the memory system, whereas in the retrieved environment, the accessibility between the two systems are equivalent.

**Table 1:** Means (standard deviations) of memory and sensorimotor aligned perspectives in different pointing directions of the three environments for both pointing latency and absolute error.

		Pointing latency			Absolute error		
Environment	Pointing direction	M	S	Comparison	M	S	Comparison
Immediate (*N* = 40)	Front	1.18 (0.69)	1.07 (0.51)	M = S	21.83 (9.24)	21.65 (9.41)	M = S
	Side	1.31 (0.81)	0.96 (0.47)	M > S	6.61 (14.05)	3.58 (6.30)	M = S
	Back	1.41 (0.60)	1.79 (1.01)	M < S	17.40 (7.03)	21.60 (10.41)	M < S
Translated (*N* = 38)	Front	1.45 (1.02)	1.49 (0.77)	M = S	20.63 (7.48)	23.56 (9.67)	M = S
	Side	1.64 (1.12)	1.36 (0.96)	M = S	3.29 (5.86)	6.93 (13.30)	M = S
	Back	1.79 (1.23)	2.53 (1.81)	M < S	17.04 (7.35)	24.18 (13.32)	M < S
Disoriented (*N* = 38)	Front	1.69 (1.28)	1.67 (0.76)	M = S	20.74 (9.18)	24.35 (13.45)	M = S
	Side	1.62 (1.03)	1.72 (1.09)	M = S	2.47 (3.43)	5.53 (9.96)	M = S
	Back	2.14 (1.01)	2.86 (1.48)	M < S	19.37 (10.13)	27.02 (15.48)	M < S


*“>” or “<” refers to significantly bigger or smaller at 0.05 level; “=” refers to no significant difference at 0.05 level. M, memory aligned perspective; S, sensorimotor aligned perspective*.

It is interesting that the representation of updating system is consistently biased away from the direction of memory aligned perspective, as if it was repelled by the representation of the memory system. This repellor effect was also observed in the study of May (2004), when participants were asked to locate target objects from some of the imagined or translated perspectives, and was suggested as the consequence of inhibition of the task-irrelevant code. Similarly, in the present experiment, when participants perform from the sensorimotor aligned perspective, the representation of memory from the learning perspective is a task-irrelevant code. It is possible that the inhibition of representation of the memory system repels the representation in the updating system. But why is representation in the updating system “repelled” away, rather than the representation in the memory system? This question needs to be further investigated.

Another interesting finding is that signed errors of misaligned perspectives are consistently biased toward the sensorimotor but not the memory aligned perspective, suggesting that the representation from the misaligned perspective is very likely being transformed from the representation in the updating but not in the memory system. Previous theories have proposed that if there are multiple encoded representations, the transformed representation should be biased toward the nearest one (Street and Wang, 2014). However, in the present experiment, the transformed representation is biased toward the farthest sensorimotor aligned perspective (180°) rather than the closest memory aligned (90°) perspective. It is possible that this proximity priority only appears when the two encoded representations are within one representation system. When the two encoded representations belong to two different systems, as in the present experiment, the selection for transformation no longer obeys the proximity priority rule. The updating system has been proposed as an online system (Waller and Hodgson, 2006) and is closely related to working memory (Priori et al., 1997; Labate et al., 2014; Piccardi et al., 2014). As spatial transformation is conducted in working memory, it is possible that the representation closer to working memory (i.e., representation in the updating system) is more preferable.

It is worth noting that besides rotation, spatial updating could also result from translation. In the immediate environment, spatial updating after translation is much easier than after rotation (e.g., Rieser, 1989; Presson and Montello, 1994), while in the retrieved environment, walking affects the encoding and updating processes differently from rotation (Hatzipanayioti et al., 2014; Santoro et al., 2017a,b). The application of bias analysis to spatial updating studies involving both rotation and translation in different environments may further our understanding of the dual systems of spatial cognition.

In summary, besides replicating the findings of memory alignment and sensorimotor alignment effects, with the analysis of pointing biases, this study further confirms that spatial memory and spatial updating systems are consistently employed across immediate and retrieved environments. Our understanding of the dual systems of spatial cognition is improved by the finding that the representation in the spatial updating system is encoded rather than transformed from that in the memory system, as it biases away rather than toward the memory aligned perspective. Additionally, our understanding is enhanced knowing that when making spatial judgments from an imagined unexperienced perspective, the representation in the updating rather than the memory system is transformed, as the judgments bias toward the sensorimotor and not the memory aligned perspective, and that the representation encoded in the updating system becomes less accessible as the environment changes from an immediate to retrieved one, which may lead to performance from sensorimotor aligned perspective becoming worse accordingly.

## Author Contributions

CL and CX are equally contributed to this article in every single detail.

## Conflict of Interest Statement

The authors declare that the research was conducted in the absence of any commercial or financial relationships that could be construed as a potential conflict of interest.

## Footnotes

1 In the retrieved environment, the absolute point error is also larger in the sensorimotor aligned than in the memory aligned perspective (Xiao et al., 2015). This could be explained as the accumulating error during updating process. As far as we know, there is no study that explicitly contrasted performance between memory aligned and sensorimotor aligned perspectives of the immediate environment; however, the equivalent pointing latency between the two perspectives could be observed in Figure 8 of the study published by Kelly et al. (2007).

2 The sensory input will be interrupted while being translated to a novel environment (e.g., Roskos-Ewoldsen et al., 1998; Wang and Brockmole, 2003; Xiao and Liu, 2014) or being disoriented (e.g., Wang and Spelke, 2000; Waller and Hodgson, 2006; Xiao et al., 2009; Xiao and Liu, 2014). In this way, participants cannot keep track of the original environment and have to retrieve the spatial representation from the memory system.
